# BSA4Yeast: Web-based quantitative trait locus linkage analysis and bulk segregant analysis of yeast sequencing data

**DOI:** 10.1093/gigascience/giz060

**Published:** 2019-05-29

**Authors:** Zhi Zhang, Paul P Jung, Valentin Grouès, Patrick May, Carole Linster, Enrico Glaab

**Affiliations:** Luxembourg Centre for Systems Biomedicine (LCSB), University of Luxembourg, 7 avenue des Hauts Fourneaux, L-4362 Esch-sur-Alzette, Luxembourg

**Keywords:** QTLs, BSA, mapping

## Abstract

**Background:**

Quantitative trait locus (QTL) mapping using bulk segregants is an effective approach for identifying genetic variants associated with phenotypes of interest in model organisms. By exploiting next-generation sequencing technology, the QTL mapping accuracy can be improved significantly, providing a valuable means to annotate new genetic variants. However, setting up a comprehensive analysis framework for this purpose is a time-consuming and error-prone task, posing many challenges for scientists with limited experience in this domain.

**Results:**

Here, we present BSA4Yeast, a comprehensive web application for QTL mapping via bulk segregant analysis of yeast sequencing data. The software provides an automated and efficiency-optimized data processing, up-to-date functional annotations, and an interactive web interface to explore identified QTLs.

**Conclusions:**

BSA4Yeast enables researchers to identify plausible candidate genes in QTL regions efficiently in order to validate their genetic variations experimentally as causative for a phenotype of interest. BSA4Yeast is freely available at https://bsa4yeast.lcsb.uni.lu.

## Background

Deciphering the genetic basis of diseases or complex traits is a major task in biomedical and basic biological research and is a key first step towards a better understanding of the molecular mechanisms behind disorders with genetic components. As a forward genetic approach, linkage analysis of quantitative trait loci (QTLs) using bulk segregant analysis (BSA) in model organisms, such as yeast, is an efficient method for identifying novel genetic variants responsible for heritable phenotypic variability [1, 2]. By exploiting the capacity of next-generation sequencing (NGS) technologies to assess large numbers of genetic markers efficiently and integrating NGS analysis with linkage mapping, the precision of QTL mapping can be improved significantly as compared to traditional approaches. In order to perform a linkage analysis using BSA in practice, relevant software packages, such as the bsaseq Python package [3], and web-based software, such as EXPLoRA-web [4], have been made available in recent years. However, these tools require researchers to first determine genetic markers of interest from sequencing data. Moreover, they only provide limited annotations for the discovered QTLs (i.e., only the QTL coordinates) and do not support the interactive exploration and visualization of detailed QTL annotations in a web browser. Because the analysis of NGS data involves several different command-line software tools and is a time-consuming and laborious task, a more efficient, automated analysis framework that supports annotation-based result interpretation would greatly facilitate NGS-based bulk segregant analysis (NGS-BSA).

**Figure 1:**
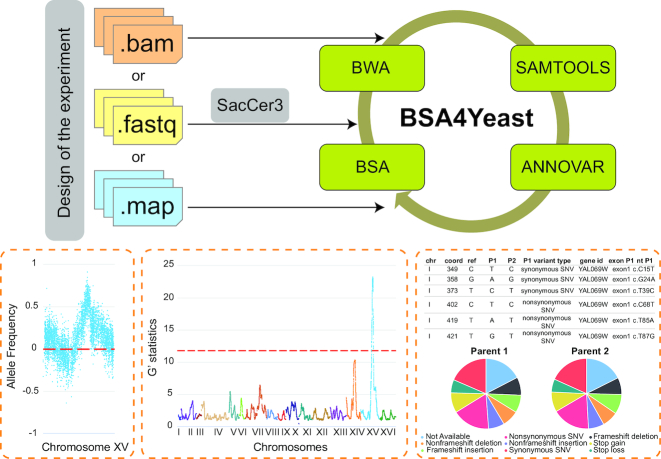
Overview of the analysis workflow for the BSA4Yeast web application. The experimental design and other parameters can be specified on the web interface. Representative results shown at the bottom include (from left to right): allele frequency, G′ statistic values, and functional annotations. SacCer3: the reference genome of *Saccharomyces cerevisiae*; SNV: single-nucleotide variant.

For this purpose, we have developed BSA4Yeast, a comprehensive web-based analysis software for QTL mapping via bulk segregant analysis of yeast sequencing data (Fig. 1). BSA4Yeast provides the following main new benefits and features:

- It enables efficient and fully automated web-based NGS-BSA without requiring prior domain expertise;
- It supports multiple input file types including .fastq, .bam, or .map (see Supplementary Methods S1, section 1 for file format explanations);
- Users can save/delete/download their analysis results and create private storage space (registration is optional and all analyses can be run without registration);
- It provides comprehensive annotations for the detected QTLs, using the latest version of the yeast reference genome. These annotations are regularly updated;
- It enables an interactive web-based exploration of the detected QTLs, their associated annotations, and statistical results.

To the best of our knowledge, BSA4Yeast is the first comprehensive web-based software that integrates automated NGS data analysis with QTL mapping via bulk segregant analysis.

## Materials and Methods

### Functionality and workflow

**Figure 2:**
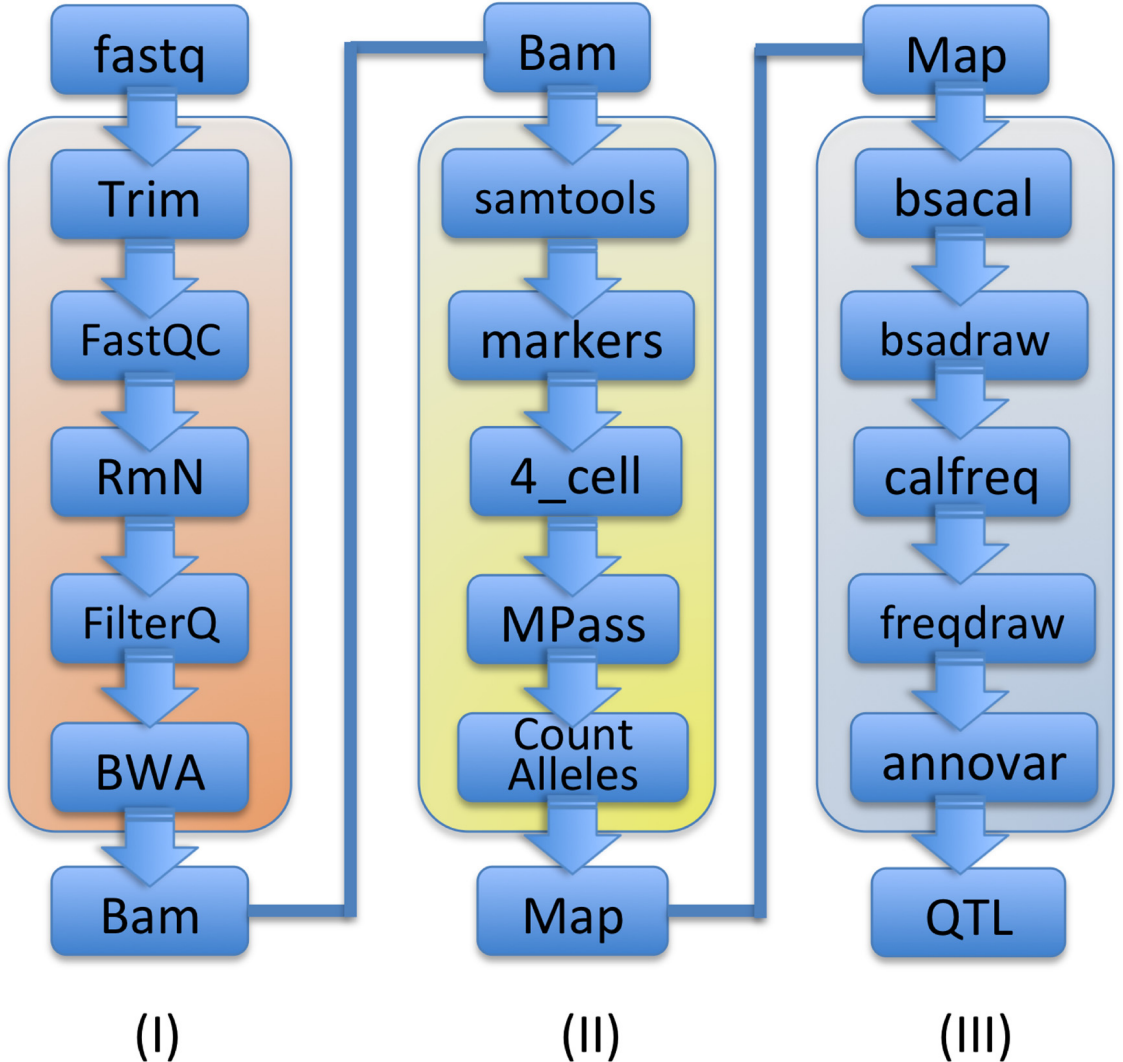
Overview of the 3 main phases of the software workflow and the individual analysis steps they include. From left to right the 3 phases cover the following tasks: (i) pre-processing and alignment of the short reads against the yeast genome, (ii) identifying genetic markers between 2 parental lines, and (iii) performing QTL analyses and comprehensively annotating the results.

The BSA4Yeast framework for QTL mapping via bulk segregant analysis of yeast sequencing data is built on custom scripts and open-source bioinformatics software (Fig. 2). The software workflow covers 3 major functionalities: (i) preprocessing and aligning short reads (Illumina format) against an up-to-date version of the yeast genome, (ii) identifying relevant genetic markers between 2 parental lines, and (iii) performing QTL analyses and comprehensively annotating the results using public data in an automated fashion (Fig. 2). For different types of experiments the design of the experiment can be adjusted appropriately using flexible parameter settings. The web application supports 1- or 2-bulk designs and designs with multiple biological replicates in each bulk, as well as 3 file formats as input (.fastq, .bam aligned against SacCer3, and .map format; both paired-end [PE] and single-end [SE] DNA sequencing data are accepted). BSA4Yeast also supports compressed input in the fastq file format (.fastq.gz). For the quality control analyses, the raw sequencing data are required; therefore, we do not use files in the Variant Call Format (VCF) or the General Feature Format (GFF) as input. However, because VCF and GFF files represent an output derived from the analysis of raw sequencing data files, the user should in most cases also have access to corresponding raw fastq or bam files, which are both accepted as input. While the default setting of BSA4Yeast is to perform BSA-QTL analyses in yeast, the experienced user can also apply the software for QTL analyses on other species, either by adding further reference genomes to the source code implementation of BSA4Yeast [5] or by creating a species-specific map file and chromosome length file as input, which requires a prior read mapping and allele count computation by the user. For adequate .map and .length input files, the public BSA4Yeast web application can be used without further changes to identify QTLs for a chosen species of interest, and for *Saccharomyces cerevisiae* dedicated annotations are generated additionally. Thus, BSA4Yeast can be used for BSA-QTL analyses either if the sequencing reads of the parental lines are provided as input or if the user only provides map files without the original sequences (which however requires a prior manual analysis of the sequencing data by the user). After the preprocessing and alignment computations in the first step of the workflow, genetic markers will be identified automatically in the second phase. Optionally, the user can adjust the trade-off between the stringency and coverage of the marker identification by specifying a custom DNA sequencing depth of coverage. For the QTL analyses in the third and final step, the user can adjust the type and width of the used smoothing kernel and has the option to download intermediate results, such as allele frequency files, bam files, or map files, for further independent analyses. The QTL peaks, QTL regions, and corresponding empirically estimated *P*-values are determined using the G′ statistic [3]. To facilitate the interpretation of the results, BSA4Yeast computes various dedicated statistics, such as the allele counts on each chromosome and a summary of the type of mutations in each parental line (e.g., stop gain, stop loss, frameshift, or non-synonymous mutations), as well as SNAP scores to evaluate deleteriousness [6]. Additionally, comprehensive annotations for the QTLs and the genome of the parental lines are provided, and all results can be downloaded from the website. The software does not require any registration, but users can optionally create an anonymous account to store results (8 GB) for a longer time to conduct further analyses with different parameters. Although BSA4Yeast cannot provide detailed quality information for each analysis step owing to the wide range of possible noise sources (experimental and technical), the multitude of quality metrics for sequencing data, and their data-dependent interpretation, if a job is not successfully completed, the software will show a guide label (yellow) on the job status page, which provides the user with indications on how to adjust the parameters. However, we also recommend that users perform their own quality control analyses for their data before uploading them to the web interface. Overall, the software is designed to enable scientists with limited background knowledge in bioinformatics to run all analysis steps with minimal manual effort, only needing to provide adequately formatted input DNA sequencing files (bam or map files) through a web browser, and avoiding time-consuming installation and configuration steps on the local computer.

### Implementation

**Figure 3:**
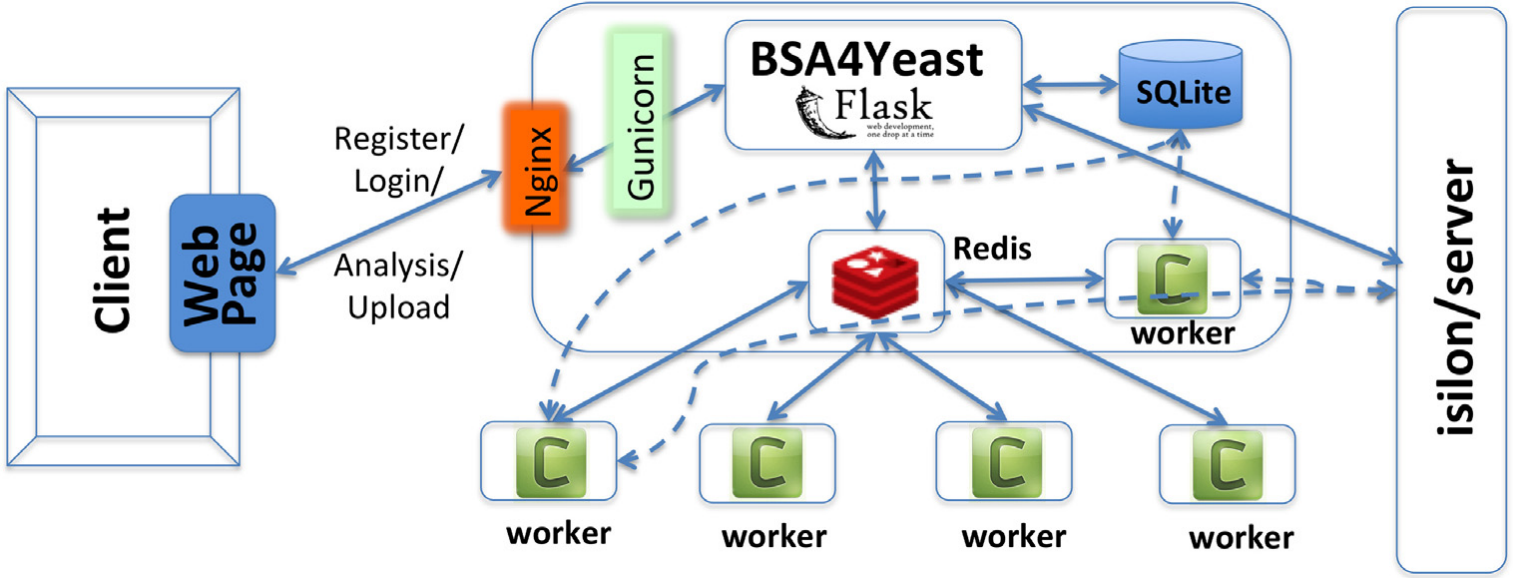
The software framework behind BSA4Yeast. The web application uses Flask and Nginx on a virtual machine, as well as Gunicorn as a Web Server Gateway Interface. Celery is employed as an asynchronous task queue/job queue system, and Redis as a message broker. The metadata for the output files are recorded in an SQLite database.

The BSA4Yeast web application has been developed in Python 2.7 using the Flask micro-framework (Fig. 3) [7]. Flask is an extensible web micro-framework, written in Python and therefore fully compatible with the BSA-sequencing package used for QTL calculations [3] (implemented in Python 2.7). All analyses run as flask asynchronous background tasks using Celery, a task queue/job queue system based on distributed messaging [8], and Redis, an open source (BSD license) message broker between the web application and the Celery worker (Fig. 3) [7, 9]. Because analyses of fastq or bam files may take hours, the user can optionally be notified about the job termination via an email message. Moreover, to avoid blocking of the main application process, analyses run as background tasks of the Celery worker (–concurrency = 4). Result files are stored on the server’s hard drive (1 TB) and periodically cleaned (a cron job every week, removing only files after a minimum waiting time of 1 week). All metadata generated when computing analysis results, such as file name, file creation time, and file type, are recorded and saved in an SQLite database. Jinja2 is used as a template language [10] and Gunicorn is employed as Web Server Gateway Interface between the web server and the web application [11]. When a job is complete or users decide to delete files for their own analyses via the web interface, the meta-information and result files will always be updated simultaneously. To allow the user to apply the web application either anonymously, or optionally, via a registered account to save results for future analyses, the application was extended by an authorization function. The overall web application is deployed with an Nginx server [12] on a dedicated virtual machine (operating system: CentOS 7.2, specifications: 16 GB RAM/8 cores).

### Tabular and visual inspection of QTL results

**Figure 4:**
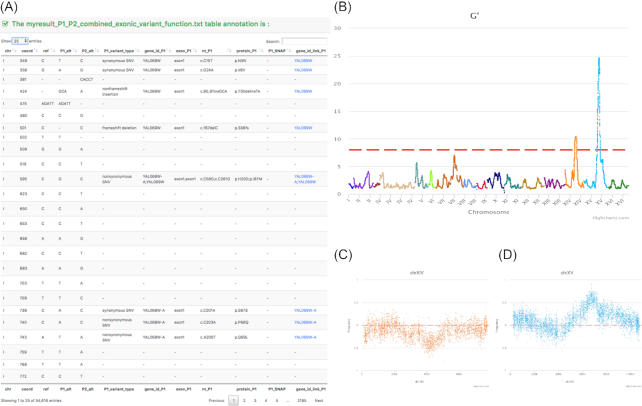
Example for visualization of QTL results: (A) annotation table for 2 parental lines (25 first entries); (B) QTL map; (C) chromosome 14 allele frequency; (D) chromosome 15 allele frequency.

**Figure 5:**
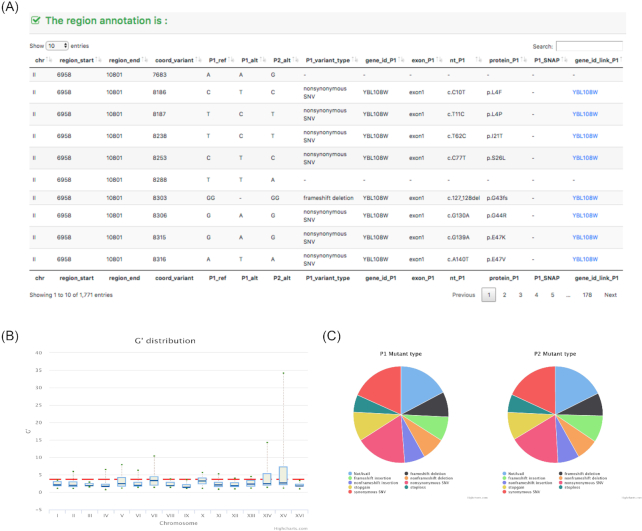
The analysis result of the fastq test file. (A) A partial annotation on QTL regions. (B) A summary of the G′ values for each chromosome. (C) A summary type of mutations from the 2 parental lines.

In order to provide an interactive and intuitive exploration of genomic data in the web browser, the BSA4Yeast graphical interface uses the libraries Bootstrap, jQuery, DataFrame.js, and Highcharts.js [13–16]. The visualization and exploration of large genomic datasets is challenging in both tabular and graphical formats. Therefore, to display large tables the dedicated library DataFrame.js is used, providing an immutable data structure supporting fast SQL queries. Moreover, we use server-side preprocessing to display a requested page, reducing the client-side computational burden. Apart from the fast retrieval of genomic information in tabular format, the annotation table is interlinked with an external yeast gene database [17], allowing the user to explore known functions for genes of interest. Visual representations of the BSA4Yeast analysis results using QTL plots and allele frequency plots can be explored dynamically using jQuery and Highcharts.js (see example in Fig. 4). Finally, pie charts for different types of mutations in each parental line can be displayed to compare their genomic diversity (see Fig. 5).

### Example application

In a first proof-of-concept study, the BSA4Yeast analysis framework was applied successfully to investigate cellular aging in baker’s yeast (*S. cerevisiae*), detecting 2 significant QTLs associated with chronological lifespan regulation [2]. Specifically, a DNA sequencing dataset consisting of PE sequenced parental strains and the SE sequenced segregant bulks was investigated with the software. The web application was applied to 3 types of input data from yeast BSA-based QTL studies, representing different experimental designs, and 2 different types of sequencing methods (PE and SE). Summary statistics for the 3 input files used for the example analyses are presented in Table 1. To test BSA4Yeast, we re-analysed 1 dataset from Jung et al. [2], which consists of the genomic DNA from 2 parent lines (YO486 and YO502) and 2 bulk segregant pools (each bulk comprising the 50 segregants with the highest or shortest chronological lifespan under low glucose concentration conditions) sequenced using 50-bp reads on an Illumina sequencer 2000 (see Supplementary Methods S1, section 2 for further details). With these data, the BSA4Yeast software recomputes and replicates the QTL results previously published by Jung et al. [2]. Representative runtimes for the example input files are ∼1 minute, ∼1.5 hours, and ∼3.5 hours for .map, .bam, and .fastq files, respectively. Example parameter settings for fastq analysis are presented in Table 2 (further example settings for other file types are provided on the BSA4Yeast website). The resulting annotation table, QTL plot, and allele frequency plot for the example analysis are shown in Fig. 4. Fig. 5 additionally displays the QTL region annotation, the G′ statistics for each chromosome, and the summarized mutation types for the 2 parental lines. Because the parental DNA data are not available when map files are used as input, only the QTL coordinates can be obtained for this input type, whereas the full annotations are generated for bam file analyses. We also created and tested a set of pseudo-mapping and pseudo-chromosome length files with a different chromosome number than for the yeast genome, to verify the applicability of the software for other genome types. All of the example datasets are available on the BSA4Yeast server for downloading and testing [18].

**Table 1.** Summary statistics for the 3 input files used for the example analyses

Format	Category	P1 (sake_strain)	P2 (white_tecc_strain)	H_bulk	L_bulk
fastq	No. of raw reads	5,669,323 (×2)	3,476,744 (×2)	10,580,579	11,915,417
bam	No. of reads aligned to reference	8,826,270	5,528,628	10,123,086	11,323,447
	Mapping rate	95.67%	94.76%	86.67%	95.70%
	Average of depth coverage	35.3	22.1	40.5	45.3
map	No. of markers			47,770	47,770

Note: ”ref” refers to the reference genome for baker’s yeast (*S. cerevisiae)*.

**Table 2.** Input parameters for analysis of fastq files

Parameter	Value
Input file type	Fastq
No. of biological replicates	1
Title for the result	myresult
1- or 2-tailed bulk design	2
The P1 Fastq file	["sake_strain_P1_1.fastq", "sake_strain_P1_2.fastq"]
The P2 Fastq file	["white_tecc_strain_P2_1.fastq", "white_tecc_strain_P2_2.fastq"]
The Bulk H_fastq file	[["Bulk_H.fastq"]]
The Bulk L_fastq file	[["Bulk_L.fastq"]]
No. of the depth of coverage	10
Type of smoothing kernel	Tricube
Width of the smoothing kernel (bp)	33,750
Chromosome number to draw	All
Whether to draw raw G′ values or not	No

## Discussion and Possible Future Extensions

BS-based QTL mapping using NGS technologies is a valuable new approach to identify genes associated with a phenotype of interest. However, the complexity of the software tools, parameter settings, and the underlying algorithms used to process the data may prevent a wider application of the computational methods developed for this purpose. Moreover, setting up a comprehensive and efficient analysis pipeline is a laborious, error-prone and time-consuming task, which requires prior experience in bioinformatics. To address these problems, speed up, and greatly facilitate bulk segregant analyses for yeast DNA sequencing data, BSA4Yeast was developed as a dedicated web application for BSA-QTL analysis. Instead of the conventional approach for QTL mapping, which investigates the allele frequency distribution across the chromosomes, BSA4Yeast uses a variant of the G-statistic [3], which provides multiple advantages over classical allele frequency analyses. First, the G-statistic is expected to decrease more rapidly around the causal site, providing narrow QTL candidate intervals; and second, the G-statistic takes into account the strength of the evidence, which is estimated using the sample size. However, certain characteristics of the G-statistic can also complicate analyses, e.g., the variance in read depth strongly influences the variance of the G-statistic over small spatial scales. The G′-statistic, a smooth version of the G-statistic, previously developed by Magwene et al. [3], is designed to address this limitation and provides a robust framework to analyse BSA sequencing data. It is computed in an automated fashion within BSA4Yeast and has been employed successfully for several biological applications, e.g., to identify genes involved in yeast biofilm formation or chronological aging [2,19]. Apart from implementing the G′-statistic for robust QTL analysis, BSA4Yeast aims at addressing some of the main hurdles in BSA-based QTL analyses discussed above both by automating and improving the efficiency of the workflow, and by facilitating the design of experiment and configuration prior to the analysis, as well as the post-analysis data interpretation, in particular for users with limited prior domain knowledge in bioinformatics. Moreover, web-based workflow implementations do not only have advantages over classical software package installations in terms of the simplicity of usage, but further benefits arise from the platform independence of the software (BSA4yeast runs on any operating system that supports modern web browsers) and the fully reproducible analyses, independent of software updates on the client’s computer. Finally, the optional password-protected access to a user account enables users to access data and results from different locations and to share them with trusted collaborators.

Because the BSA4Yeast workflow is implemented in a modular fashion, it can be extended and adjusted, e.g., to cover further annotations and reference genomes for other model organisms used to perform linkage QTL studies (e.g., fruit flies or mice). Moreover, the software can be interlinked with other public internet databases and repositories, which contain further information on identified genes with a phenotype association of interest. The BSA4Yeast source code has been made available on GitLab [5] to allow other users to explore, modify, or further extend the software. The first author (Z.Z.) will continue to maintain the website interface and provide technical support and updates for the users.

## Summary

In summary, BSA4Yeast was designed and implemented to enable users to perform comprehensive NGS-BSA studies efficiently without requiring prior bioinformatics knowledge. The overall software workflow (Fig. 1) covers the following data processing, quality control, and analysis tasks:

1. Mapping short reads (PE or SE, Illumina format) to the standard *S. cerevisiae* reference genome (UCSC release SacCer3; BWA, version 0.7.4) [20] and generating alignment files (.bam format) for both parental and bulk samples;
2. Defining genetic markers between 2 parental lines given a user-defined coverage threshold (default: 5×);
3. Calculating G′ statistical values [3] for each genetic marker in the bulk pools;
4. Annotating exonic variants between the 2 parental lines and within each QTL region with ANNOVAR (version: Mon, 1 Feb 2016) [21];
5. Scoring the functional impact of non-synonymous variants with SNAP (version: 2.0) [6].

The final results generated by the software, including the annotations, variant functional impact predictions, and statistical results for each determined QTL region, can be explored interactively and downloaded using current standard web browsers (tested on Chrome, Firefox, and Safari).

## Availability of source code and requirements

- Project name: BSA4Yeast
- RRID: SCR_017113
- Project home page: https://git-r3lab.uni.lu/zhi.zhang/bsa4yeast
- Operating system(s): CentOS 7.2
- Programming language: Python 2.7
- Other requirements: Flask 0.12.2, Celery 4.1.0, Redis 2.10.6
- License: GNU GPL

## Availability of supporting data and materials

The example datasets presented are available at https://bsa4yeast.lcsb.uni.lu. All other supporting data are also available via the *GigaScience* GigaDB repository [22]. For more information on the experimental design and statistical methods used in BSA4Yeast, please see the Supplementary Materials.

## Additional files

Supplementary Methods S1. File format descriptions, experimental design, sequencing data processing and statistical methods.

## Abbreviations

bp: base pair; BSA: bulk segregant analysis; GB: gigabytes; GFF: General Feature Format; GPL: General Public License; HPC: high-performance computing; NGS: next-generation sequencing; PE: paired end; RAM: random access memory; QTL: quantitative trait locus; SE: single end; SNV: single-nucleotide variant; SQL: Structured Query Language; UCSC: University of California Santa Cruz; VCF: Variant Call Format.

## Competing interests

The authors declare that they have no competing interests.

## Funding

Acknowledgement is made for support by the Fonds Nationale de la Recherche (FNR) Luxembourg, through the National Centre of Excellence in Research (NCER) on Parkinson’s disease (I1R-BIC-PFN-15NCER), and as part of the grant project PD-Strat (INTER/11651464).

## Authors' contributions

Z.Z. and E.G. designed the project. Z.Z. wrote the code of the project. Z.Z., P.P.J., and E.G. wrote the manuscript. V.G. contributed his knowledge to the server infrastructure and deployment. P.M. provided the SNAP2.0 value for mutations. P.P.J. and C.L. contributed the sequencing datasets for testing. All authors read the manuscript and provided feedback.

## Supplementary Material

giz060_GIGA-D-18-00409_Original_Submission

giz060_GIGA-D-18-00409_Revision_1

giz060_GIGA-D-18-00409_Revision_2

giz060_GIGA-D-18-00409_Revision_3

giz060_Response_to_Reviewer_Comments_Original_Submission

giz060_Response_to_Reviewer_Comments_Revision_1

giz060_Response_to_Reviewer_Comments_Revision_2

giz060_Reviewer_1_Report_Original_Submission -- Christian Brion, Ph.D, 12/8/2018 Reviewed

giz060_Reviewer_1_Report_Revision_1 -- Christian Brion, Ph.D, 3/4/2019 Reviewed

giz060_Reviewer_2_Report_Original_Submission -- Francisco Cubillos 12/11/2018 Reviewed

giz060_Supplemental_File
