# Supplementary material for: BSA4Yeast: Web-based quantitative trait locus linkage analysis and bulk segregant analysis of yeast sequencing data
Source: Gigascience. 2019 May 29;8(6):giz060. doi: 10.1093/gigascience/giz060 (PMC6571488; doi:10.1093/gigascience/giz060)
Supplement: giz060_GIGA-D-18-00409_Revision_2 [file giz060_giga-d-18-00409_revision_2.pdf]

GigaScience

BSA4Yeast: Web-based QTL linkage analysis and bulk segregant analysis of yeast sequencing data

--Manuscript Draft--

**Manuscript Number:**	GIGA-D-18-00409R2
**Full Title:**	BSA4Yeast: Web-based QTL linkage analysis and bulk segregant analysis of yeast sequencing data
**Article Type:**	Technical Note
**Funding Information:**	Fonds National de la Recherche Luxembourg (I1R-BIC-PFN-15NCER) — Not applicable<br>Fonds National de la Recherche Luxembourg (INTER/11651464) — Not applicable
**Abstract:**	Background: Quantitative Trait Loci (QTL) mapping using bulk segregants is an effective approach for identifying genetic variants associated with phenotypes of interest in model organisms. By exploiting next-generation sequencing technology, the QTL mapping accuracy can be improved significantly, providing a valuable means to annotate new genetic variants. However, setting up a comprehensive analysis framework for this purpose is a time-consuming and error prone task, posing many challenges for scientists with limited experience in this domain.<br><br>Findings: Here, we present BSA4Yeast, a comprehensive web-application for QTL mapping via bulk segregant analysis of yeast sequencing data. The software provides an automated and efficiency-optimized data processing, up-to-date functional annotations, and an interactive web-interface to explore identified QTLs.<br><br>Conclusion: BSA4Yeast enables researchers to identify plausible candidate genes in QTL regions efficiently in order to experimentally validate causative genetic variations for a phenotype of interest. BSA4Yeast is freely available at https://bsa4yeast.lcsb.uni.lu.
**Corresponding Author:**	Enrico Glaab<br>University of Luxembourg<br>Esch-sur-Alzette, Capellen LUXEMBOURG
**Corresponding Author Secondary Information:**	
**Corresponding Author's Institution:**	University of Luxembourg
**Corresponding Author's Secondary Institution:**	
**First Author:**	Zhi Zhang
**First Author Secondary Information:**	
**Order of Authors:**	Zhi Zhang<br>Paul P. Jung<br>Valentin Grouès<br>Patrick May<br>Carole Linster<br>Enrico Glaab
**Order of Authors Secondary Information:**	
**Response to Reviewers:**	Dear Editor,<br><br>we thank you and the reviewers for the efficient handling of the manuscript. We have

	addressed the three final editorial issues mentioned in the response letter as follows:<br><br>1) GigaDB repository: We have included a citation of the upcoming GigaDB dataset (reference 19) - please note that the DOI number was not yet available, but we have been informed by Chris Armit that the GigaScience Editors will add the final DOI number to template DOI link in our manuscript.<br><br>2) All highlighting / tracking of changes for the reviewers has been removed from the final manuscript.<br><br>3) Our software application has been registered in the SciCrunch.org database and the RRID has been added to the manuscript. (the RRID-link is expected to become active after the 20th of April).<br><br>Yours sincerely,<br>Enrico Glaab
**Additional Information:**	
**Question**	**Response**
Are you submitting this manuscript to a special series or article collection?	No
**Experimental design and statistics**<br><br>Full details of the experimental design and statistical methods used should be given in the Methods section, as detailed in our Minimum Standards Reporting Checklist. Information essential to interpreting the data presented should be made available in the figure legends.<br><br>Have you included all the information requested in your manuscript?	Yes
**Resources**<br><br>A description of all resources used, including antibodies, cell lines, animals and software tools, with enough information to allow them to be uniquely identified, should be included in the Methods section. Authors are strongly encouraged to cite Research Resource Identifiers (RRIDs) for antibodies, model organisms and tools, where possible.<br><br>Have you included the information requested as detailed in our Minimum Standards Reporting Checklist?	Yes

Powered by Editorial Manager® and ProduXion Manager® from Aries Systems Corporation

**Availability of data and materials**<br><br>All datasets and code on which the conclusions of the paper rely must be either included in your submission or deposited in publicly available repositories (where available and ethically appropriate), referencing such data using a unique identifier in the references and in the "Availability of Data and Materials" section of your manuscript.<br><br>Have you have met the above requirement as detailed in our Minimum Standards Reporting Checklist?	Yes

Manuscript
Click here to view linked References
Click here to access/download;Manuscript;bsa4yeast_revised_manuscript.t

OXFORD
(GIGA)^n SCIENCE

GigaScience, 2018, 1–7

doi: xx.xxxx/xxxx
Manuscript in Preparation
Technical note

TECHNICAL NOTE

BSA4Yeast: Web-based QTL linkage analysis and bulk segregant analysis of yeast sequencing data

Zhi Zhang[1], Paul P Jung[1], Valentin Groués[1], Patrick May[1], Carole Linster[1] and Enrico Glaab[1,*]

[1]Luxembourg Centre for Systems Biomedicine (LCSB), University of Luxembourg, Esch-sur-Alzette, Luxembourg

[*]enrico.glaab@uni.lu

Abstract

Background: Quantitative Trait Loci (QTL) mapping using bulk segregants is an effective approach for identifying genetic variants associated with phenotypes of interest in model organisms. By exploiting next-generation sequencing technology, the QTL mapping accuracy can be improved significantly, providing a valuable means to annotate new genetic variants. However, setting up a comprehensive analysis framework for this purpose is a time-consuming and error prone task, posing many challenges for scientists with limited experience in this domain. **Findings:** Here, we present BSA4Yeast, a comprehensive web-application for QTL mapping via bulk segregant analysis of yeast sequencing data. The software provides an automated and efficiency-optimized data processing, up-to-date functional annotations, and an interactive web-interface to explore identified QTLs. **Conclusion:** BSA4Yeast enables researchers to identify plausible candidate genes in QTL regions efficiently in order to validate their genetic variations experimentally as causative for a phenotype of interest. BSA4Yeast is freely available at https://bsa4yeast.lcsb.uni.lu.

Key words: QTLs; BSA; mapping

Background

Deciphering the genetic basis of diseases or complex traits is a major task in biomedical and basic biological research and is a key first step towards a better understanding of the molecular mechanisms behind disorders with genetic components. As a forward genetic approach, linkage analysis of Quantitative Trait Loci (QTLs) using bulk segregant analysis (BSA) in model organisms, such as yeast, is an efficient method for identifying novel genetic variants responsible for heritable phenotypic variability [1, 2]. By exploiting the capacity of next-generation sequencing (NGS) technologies to assess large numbers of genetic markers efficiently and integrating NGS analysis with linkage mapping, the precision of QTL mapping can be improved significantly as compared to traditional approaches. In order to perform a linkage analysis using BSA in practice, relevant software packages, such as the *bsaseq* python package [3], and web-based software, such as *EXPLoRA-web* [4], have been made available in recent years. However, these tools require researchers to first determine genetic markers of interest from sequencing data. Moreover, they only provide limited annotations for the discovered QTLs (i.e. only the QTL coordinates) and do not support the interactive exploration and visualization of detailed QTL annotations in a web-browser. Since the analysis of NGS data involves several different command-line software tools and is a time-consuming and laborious task, a more efficient, automated analysis framework that supports annotation-based result interpretation would greatly facilitate NGS-based bulk segregant analysis (NGS-BSA).

For this purpose, we have developed BSA4Yeast, a comprehensive web-based analysis software for QTL mapping via bulk segregant analysis of yeast sequencing data (Fig. 1). BSA4Yeast provides the following main new benefits and features:

- It enables efficient and fully automated web-based NGS-BSA without requiring prior domain expertise;

Compiled on: April 17, 2019.
Draft manuscript prepared by the author.

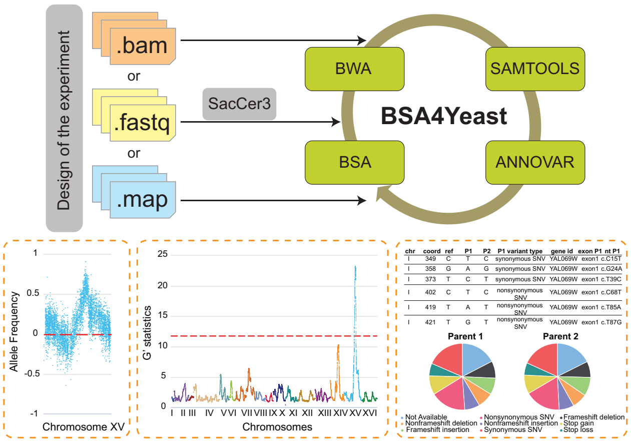

Figure 1. Overview of the analysis workflow for the BSA4Yeast web-application. The experimental design and other parameters can be specified on the web-interface. Representative results shown at the bottom include (from left to right): allele frequency, G' statistic values and functional annotations. SacCer3: the reference genome of *Saccharomyces cerevisiae*.

- It supports multiple input file types including .fastq, .bam or .map (see Suppl. Mat. for file format explanations);
- Users can save/delete/download their analysis results and create private storage space (registration is optional and all analyses can be run without registration);
- It provides comprehensive annotations for the detected QTLs, using the latest version of the yeast reference genome. These annotations are regularly updated;
- It enables an interactive web-based exploration of the detected QTLs, their associated annotations and statistical results.

To the best of our knowledge, BSA4Yeast is the first comprehensive web-based software that integrates automated NGS data analysis with QTL mapping via bulk segregant analysis.

Materials and Methods

Functionality and Workflow

The BSA4Yeast framework for QTL mapping via bulk segregant analysis of yeast sequencing data is built on custom scripts and open-source bioinformatics software (Fig. 2).The software workflow covers three major functionalities: 1) Pre-processing and aligning short reads (Illumina format) against an up-to-date version of the yeast genome; 2) identifying relevant genetic markers between two parental lines; and 3) performing QTL analyses and comprehensively annotating the results using public data in an automated fashion (Fig. 2). For different types of experiments the design of the experiment (DOE) can be adjusted appropriately using flexible parameter settings. The web-application supports one- or two-bulk designs and designs with multiple biological replicates in each bulk, as well as three file formats as input (.fastq, .bam aligned against SacCer3, and .map format; both paired-end (PE) or single-end (SE) DNA sequencing data is accepted). BSA4Yeast also supports compressed input in the fastq file format (.fastq.gz). For the quality control analyses, the raw sequencing data is required, therefore we do not use files in the Variant Call Format (VCF) or the General Feature Format (GFF) as input. However, since VCF- and GFF-files represent an output derived from the analysis of raw sequencing data files, the user should in most cases also have access to corresponding raw fastq- or bam-files, which are both accepted as input. While the default setting of BSA4Yeast is to perform BSA-QTL analyses in yeast, the experienced user can also apply the software for QTL analyses on other species, either by adding further reference genomes to the source code implementation of BSA4Yeast (available at https://git-r3lab.uni.lu/zhi.zhang/bsa4yeast) or by creating a species-specific map file and chromosome length file as input, which requires a prior read mapping and allele count computation by the user. For adequate .map and .length input files, the public BSA4Yeast web-application can be used without further changes to identify QTLs for a chosen species of interest, and for *S. cerevisiae* dedicated annotations are generated additionally. Thus, BSA4Yeast can be used for BSA-QTL analyses either if the sequencing reads of the parental lines are provided as input, or if the user only provides map-files without the original sequences (which however requires a prior manual analysis of the sequencing data by the user). After the pre-processing and alignment computations in the first step of the workflow, genetic markers will be identified automatically in the second phase. Optionally, the user can adjust the trade-off between the stringency and coverage of the marker identification by specifying a custom DNA sequencing depth of coverage. For the QTL analyses in the third and final step, the user can adjust the type and width of the used smoothing kernel and has the option to download intermediate results, such as allele frequency files, bam files or map files, for further independent analyses. The QTL peaks, QTL regions and corresponding empirically estimated p-values are determined using the G' statistic [3]. To facilitate the results interpretation, BSA4Yeast computes various dedicated statistics, such as the allele counts on each chromosome and a summary of the type of mutations in each parental line (e.g. stop gain, stop loss, frameshift or nonsynonymous mutations), as well as SNAP scores to evaluate deleteriousness [5]. Additionally, comprehensive annotations for the QTLs and the genome of the parental lines are provided, and all results can be downloaded from the website. The software does not require any registration, but users can optionally create an anonymous account to store results (8 GB) for a longer time to conduct further analyses with different parameters. Although BSA4Yeast cannot provide detailed quality information for each analysis step due to the wide range of possible noise sources (experimental and technical), the multitude of quality metrics for sequencing data and their data-dependent interpretation, if a job is not successfully completed, the software will show a guide label (yellow) on the job status page, which provides the user with indications on how to adjust the parameters. However, we also recommend that users perform their own quality control analyses for their data before uploading it on the web-interface. Overall, the software is designed to enable scientists with limited background knowledge in bioinformatics to run all analysis steps with minimal manual effort, only needing to provide adequately formatted input DNA sequencing files (bam or map files) through a web-browser, and avoiding time-consuming installation and configuration steps on the local computer.

Implementation

The BSA4Yeast web-application has been developed in Python 2.7 using the Flask micro-framework (Fig. 3) [6]. Flask is an extensible web micro-framework, written in Python and therefore fully compatible with the bsa-seq package used for QTL calculations [3] (implemented in Python 2.7). All analyses run as flask asynchronous background tasks using Celery, a task queue/job queue system based on distributed messaging [7], and Redis, an open source (BSD license) message broker between the web-application and the celery worker (Fig. 3) [8, 6]. Since analyses of fastq or bam files may take hours, the user can optionally be notified about the job termination via

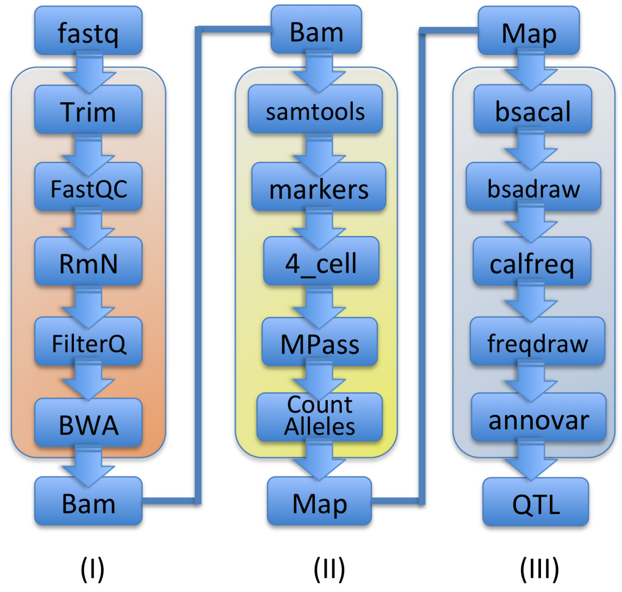

Figure 2. Overview of the three main phases of the software workflow and the individual analysis steps they include. From left to right the three phases cover the following tasks: (I) pre-processing and alignment of the short reads against the yeast genome; (II) identifying genetic markers between two parental lines; and (III) performing QTL analyses and comprehensively annotating the results.

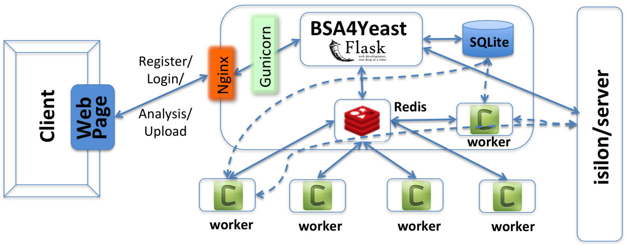

Figure 3. The software framework behind BSA4Yeast. The web-application uses Flask and Nginx on a virtual machine, as well as Gunicorn as a Web Server Gateway Interface. Celery is employed as an asynchronous task queue/job queue system, and redis as a message broker. The metadata for the output files is recorded in an SQLite database.

an email message. Moreover, to avoid blocking of the main application process, analyses run as background tasks of the Celery worker (–concurrency = 4). Result files are stored on the server's hard drive (1 TB) and periodically cleaned (a cron job every week, removing only files after a minimum waiting time of one week). All metadata generated when computing analysis results, such as file name, file creation time, file type, is recorded and saved in an SQLite database. Jinja2 is used as a template language [9] and Gunicorn is employed as Web Server Gateway Interface between the web-server and the web-application [10]. When a job is complete or users decide to delete files for their own analyses via the web-interface, the meta-information and result files will always be updated simultaneously. To allow the user to apply the web-application either anonymously, or optionally, via a registered account to save results for future analyses, the application was extended by an authorization function. The overall web-application is deployed with an Nginx server [11] on a dedicated virtual machine (operating system: CentOS 7.2, specifications: 16 GB RAM / 8 cores).

Tabular and visual inspection of QTL results

In order to provide an interactive and intuitive exploration of genomic data in the web-browser, the BSA4Yeast graphical interface uses the libraries Bootstrap, jQuery, DataFrame.js and Highcharts.js [12, 13, 14, 15]. The visualization and exploration of large genomic datasets is challenging in both tabular and graphical formats. Therefore, to display large tables the dedicated library DataFrame.js is used, providing an immutable data structure supporting fast SQL queries. Moreover, we use server-side pre-processing to display a requested page, reducing the client side computational burden. Apart from the fast retrieval of genomic information in tabular format, the annotation table is interlinked with an external yeast gene database (SGD: `https://www.yeastgenome.org`), allowing the user to explore known functions for genes of interest. Visual representations of the BSA4Yeast analysis results using QTL plots and allele frequency plots can be explored dynamically using jQuery and Highcharts.js (see example in Fig. 4). Finally, pie charts for different types of mutations in each parental line can be displayed to compare their genomic diversity (see Fig. 5).

Example application

In a first proof-of-concept study, the BSA4Yeast analysis framework was applied successfully to investigate cellular aging in baker's yeast (*S. cerevisiae*), detecting two significant QTLs associated with chronological life span regulation [2]. Specifically, a DNA sequencing dataset consisting of paired end (PE) sequenced parental strains and the single end (SE) sequenced segregant bulks was investigated with the software. The web-application was applied on three types of input data from yeast BSA-based QTL studies, representing different experimental designs, and two different types of sequencing methods (PE and SE). Summary statistics for the three input files used for the example analyses are shown in Table 1. To test BSA4Yeast, we re-analysed one dataset from Jung et al. (2018), which consists of the genomic DNA from two parent lines (YO486 and YO502) and two bulk segregant pools (each bulk comprising the 50 segregants with the highest or shortest chronological life span under low glucose conditions) sequenced using 50-bp reads on an Illumina sequencer 2000 (see the Suppl. Mat. for further details). With this data, the BSA4Yeast software recomputes and replicates the QTL results previously published by Jung et al. (2018) [2]. Representative runtimes for the example input files are ~1 min, ~1.5 hours and ~3.5 hours for .map, .bam and .fastq files. Example parameter settings for fastq analysis are shown in Table 2 (further example settings for other file types are provided on the BSA4Yeast web-site). The resulting annotation table, QTL plot and allele frequency plot for the example analysis are shown in Fig. 4. Fig. 5 additionally displays the QTL region annotation, the G' statistics for each chromosome and the summarized mutation types for the two parental lines. Since the parental DNA data is not available when using map files as input, only the QTL coordinates can be obtained for this input type, whereas the full annotations are generated for bam-file analyses. We also created and tested a set of pseudo-mapping and pseudo-chromosome length files with a different chromosome number than for the yeast genome, to verify the applicability of the software for other genome types. All of the example datasets are available on the BSA4Yeast server for downloading and testing (https://bsa4yeast.lcsb.uni.lu/).

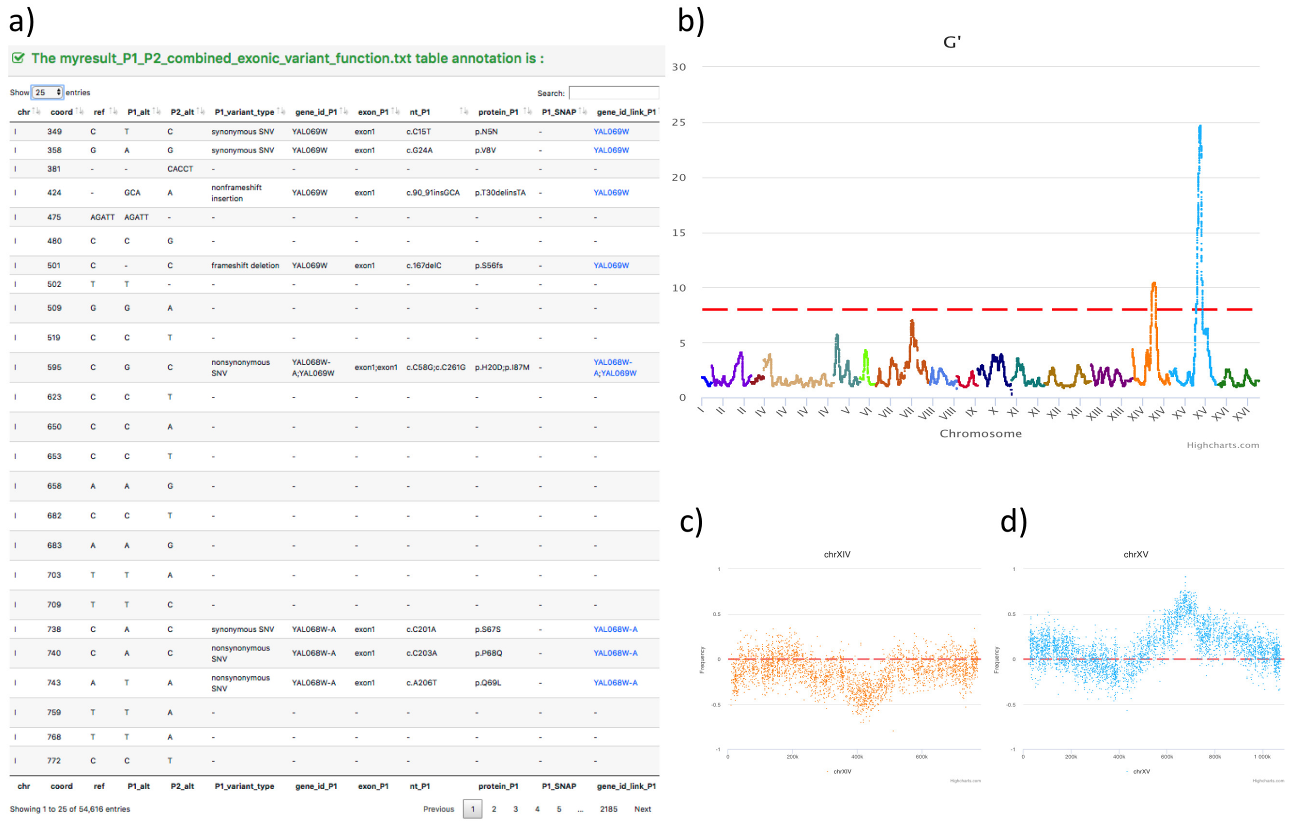

Figure 4. Example for visualization of QTL results: a) Annotation table for two parental lines (25 first entries); b) QTL map; c) Chromosome 14 allele frequency; d) Chromosome 15 allele frequency.

Table 1. Summary statistics for the three input files used for the example analyses.

Format	category	P1 (sake_strain)	P2 (white_tecc_strain)	H_bulk	L_bulk
fastq	no. of raw reads	5669323(×2)	3476744(×2)	10580579	11915417
bam	no. of reads aligned to ref	8826270	5528628	10123086	11323447
	mapping rate	95,67%	94,76%	86.67%	95.70%
	average of depth coverage	35,3	22,1	40.5	45.3
map	number of markers	-	-	47770	47770

Note: "ref" refers to the reference genome for baker's yeast (*S. cerevisiae).*

Discussion and possible future extensions

Bulk Segregant Analysis (BSA) based QTL mapping using next generation sequencing technologies is a valuable new approach to identify genes associated with a phenotype of interest. However, the complexity of the software tools, parameter settings and the underlying algorithms used to process the data may prevent a wider application of the computational methods developed for this purpose. Moreover, setting up a comprehensive and efficient analysis pipeline is a laborious, error-prone and time-consuming task, which requires prior experience in bioinformatics. To address these problems, speed up and greatly facilitate bulk segregant analyses for yeast DNA sequencing data, BSA4Yeast was developed as a dedicated web-application for BSA-QTL analysis. Instead of the conventional approach for QTL mapping, which investigates the allele frequency distribution across the chromosomes, BSA4Yeast uses a variant of the G-statistic [3], which provides multiple advantages over classical allele frequency analyses. Firstly, the G-statistic is expected to decrease more rapidly around the causal site, providing narrow QTL candidate intervals; and secondly, the G-statistic takes into account the strength of the evidence, which is estimated using the sample size. However, certain characteristics of the G-statistic can also complicate analyses, e.g. the variance in read depth strongly influences the variance of the G-statistic over small spatial scales. The G'-statistic, a smooth version of the G-statistic, previously developed by Paul Magwene et al. [3], is designed to address this limitation and provides a robust framework to analyze BSA sequencing data. It is computed in an automated fashion within BSA4Yeast and has been employed successfully for several biological applications, e.g. to identify genes involved in yeast biofilm formation or chronological aging [2, 16]. Apart from implementing the G'-statistic for robust QTL analysis, BSA4Yeast aims at addressing some of the main hurdles in BSA-based QTL analyses discussed above both by automating and improving the efficiency of the workflow, and by facilitating the design of experiment and configuration prior to the analysis, as well as the post-analysis data interpretation, in particular for users with limited prior domain knowledge in bioinformatics. Moreover, web-based workflow implementations do not only have advantages over classical software package installations in terms of the simplicity of usage, but further benefits arise from the platform-independence of the software (BSA4yeast runs on any operating system that supports modern web-browsers) and the fully reproducible analyses, independent of software updates on the

Table 2. Input parameters for analysis of fastq files.

Parameters	Value
Input file type	Fastq
Number of biological replicates	1
Title for the result	myresult
One- or two-tailed bulk design	2
The P1 Fastq file	['sake_strain_P1_1.fastq', 'sake_strain_P1_2.fastq']
The P2 Fastq file	['white_tecc_strain_P2_1.fastq', 'white_tecc_strain_P2_2.fastq']
The Bulk H_fastq file	[['Bulk_H.fastq']]
The Bulk L_fastq file	[['Bulk_L.fastq']]
The number of the depth of coverage	10
The type of smoothing kernel	tricube
The width of the smoothing kernel (bp)	33750
The chromosome number to draw	all
Whether to draw raw G' values or not	no

client's computer. Finally, the optional password-protected access to a user account enables users to access data and results from different locations and to share them with trusted collaborators.

Since the BSA4Yeast workflow is implemented in a modular fashion, it can be extended and adjusted, e.g. to cover further annotations and reference genomes for other model organisms used to perform linkage QTL studies (e.g. fruit flies or mice). Moreover, the software can be interlinked with other public internet databases and repositories, which contain further information on identified genes with a phenotype association of interest. The BSA4Yeast source code has been made available on GitLab (https://git-r3lab.uni.lu/zhi.zhang/bsa4yeast) to allow other users to explore, modify or further extend the software. The first author, Zhi Zhang, will continue to maintain the website interface, and provide technical support and updates for the users.

Summary

In summary, BSA4Yeast was designed and implemented in order to enable users to perform comprehensive NGS-BSA studies efficiently without requiring prior bioinformatics knowledge. The overall software workflow (Fig. 1) covers the following data processing, quality control and analysis tasks:

i. Mapping short reads (PE or SE, Illumina format) to the standard *Saccharomyces cerevisiae* reference genome (UCSC release SacCer3; BWA, version 0.7.4) [17] and generating alignment files (.bam format) for both parental and bulk samples;

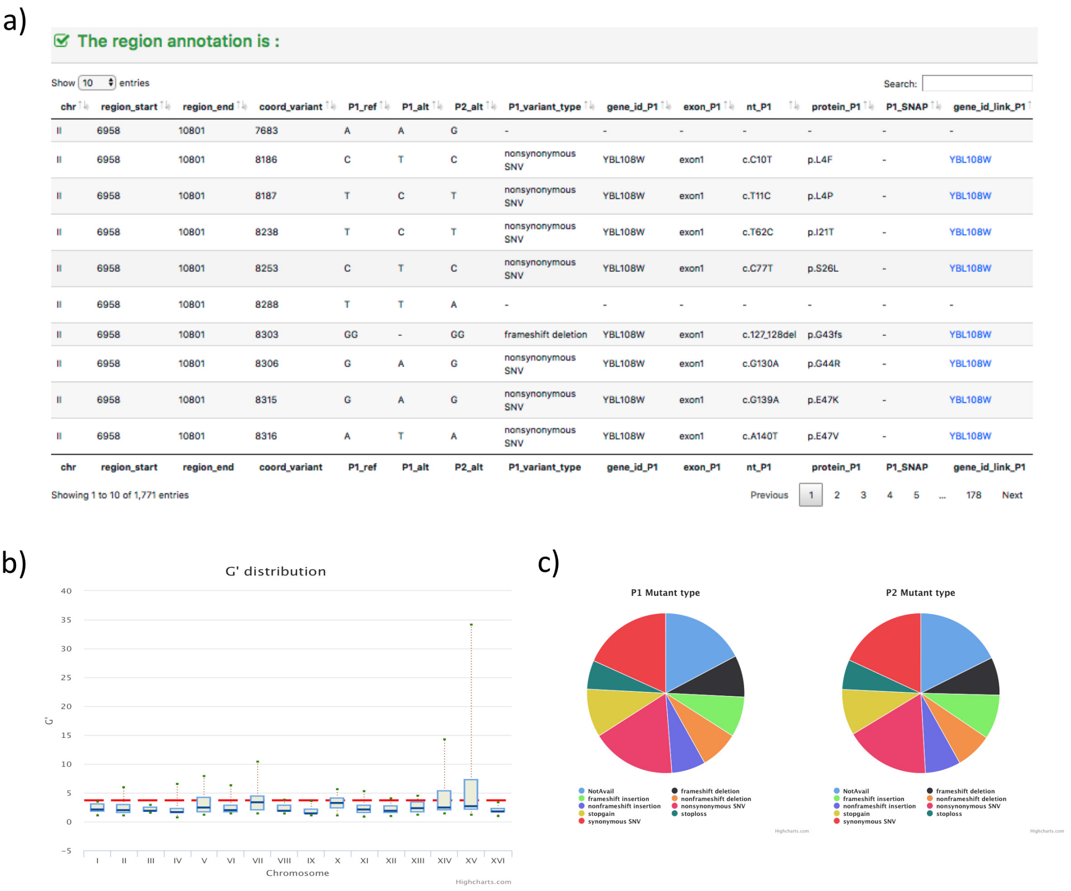

Figure 5. The analysis result of the fastq test file. a) A partial annotation on QTL regions. b) A summary of the G' values for each chromosome, c) A summary type of mutations from the two parental lines.

ii. Defining genetic markers between two parental lines given a user-defined coverage threshold (default: 5×);
iii. Calculating G' statistical values [3] for each genetic marker in the bulk pools;
iv. Annotating exonic variants between the two parental lines and within each QTL region with ANNOVAR (version: Mon, 1 Feb 2016) [18];
v. Scoring the functional impact of non-synonymous variants with SNAP (version: 2.0) [5].

The final results generated by the software, including the annotations, variant functional impact predictions and statistical results for each determined QTL region, can be explored interactively and downloaded using current standard web-browsers (tested on Chrome, Firefox and Safari).

Availability of source code and requirements

- Project name: BSA4Yeast
- RRID: SCR_017113
- Project home page: https://git-r3lab.uni.lu/zhi.zhang/bsa4yeast
- Operating system(s): CentOS 7.2
- Programming language: Python 2.7
- Other requirements: Flask 0.12.2, Celery 4.1.0, Redis 2.10.6
- License: GNU GPL

Availability of supporting data and materials

The used example datasets are available at https://bsa4yeast.lcsb.uni.lu. All other supporting data are also available via the GigaScience GigaDB repository [19]. For more information on the experimental design and statistical methods used in BSA4Yeast, please see the Supplementary Information.

Declarations

List of abbreviations

- BSA: Bulk Segregant Analysis
- DOE: Design of Experiment
- NGS: Next Generation Sequencing
- PE: Paired-end
- QTL: Quantitative Trait Locus
- SE: Single-end
- VM: Virtual Machine
- web-app: web application
- WSGI: Web Server Gateway Interface

Consent for publication

Not applicable

Competing Interests

The authors declare(s) that they have no competing interests.

Funding

Acknowledgement is made for support by the Fonds Nationale de la Recherche (FNR) Luxembourg, through the National Centre of Excellence in Research (NCER) on Parkinson's disease (I1R-BIC-PFN-15NCER), and as part of the grant project PD-Strat (INTER/11651464).

Author's Contributions

ZZ, EG designed the project. ZZ wrote the code of project. ZZ, PJ, EG wrote the manuscript. VG contributed his knowledge to the server infrastructure and deployment. PM provided the SNAP2.0 value for mutations. PJ, CL contributed the sequencing data sets for testing. All authors read the manuscript and provided feedback.

Acknowledgements

We thank Yohan Jarosz, Roland Krause and Ursula Heins-Marroquin for advice and helpful discussions, Venkata Satagopam for the calculated SNAP scores, Maharashi Yvas for help with the server backup, and Paul Magwene for kindly providing source code used for the BSA implementation. The bioinformatics analyses presented in this paper were partly conducted using the HPC facilities at the University of Luxembourg (see http://hpc.uni.lu).

References

1. Wilkening S, Lin G, Fritsch ES, Tekkedil MM, Anders S, Kuehn R, et al. An evaluation of high-throughput approaches to QTL mapping in Saccharomyces cerevisiae. Genetics 2014 Mar;196:853–865.
2. Jung PP, Zhang Z, Paczia N, Jaeger C, Ignac T, May P, et al. Natural variation of chronological aging in the Saccharomyces cerevisiae species reveals diet-dependent mechanisms of life span control. NPJ Aging and Mechanisms of Disease 2018;4:3.
3. Magwene PM, Willis JH, Kelly JK. The statistics of bulk segregant analysis using next generation sequencing. PLoS Computational Biology 2011 Nov;7:e1002255.
4. Pulido-Tamayo S, Duitama J, Marchal K. EXPLoRA-web: linkage analysis of quantitative trait loci using bulk segregant analysis. Nucleic Acids Research 2016 Jul;44:W142–W146.
5. Bromberg Y, Rost B. SNAP: predict effect of non-synonymous polymorphisms on function. Nucleic Acids Research 2007;35:3823–3835.
6. Grinberg M. Flask web development. O'Reilly Media; 2018.
7. Sileika R. Pro Python System Administration. Springer; 2014.
8. Macedo T, Oliveira F. Redis Cookbook. O'Reilly Media; 2011.
9. Rubio D. Beginning Django. Berkeley: Apress; 2017.
10. Gardner J. The Definitive Guide to Pylons. Berkeley: Apress; 2009.
11. Soni R. Nginx. Berkeley: Apress; 2016.
12. Wang Y, Wang J. Application of Highcharts in the Analysis of the Multi-source Track Inspection Data. International Conference on Mechatronics, Electronic, Industrial and Control Engineering (MEIC 2015), Atlantis Press; 2015. p. 1533.
13. Budd A, Björklund E. CSS Mastery. Berkeley: Apress; 2016.
14. Freeman A. Pro AngularJS. Berkeley: Apress; 2014.
15. Chaudhary M, Kumar A. Practical jQuery. Berkeley: Apress; 2015.
16. Granek JA, Murray D, Kayıkçı Ö, Magwene PM. The genetic architecture of biofilm formation in a clinical isolate of Saccharomyces cerevisiae. Genetics 2012;p. genetics–112.
17. Li H, Durbin R. Fast and accurate short read alignment

with Burrows-Wheeler transform. Bioinformatics (Oxford, England) 2009 Jul;25:1754–1760.
18. Wang K, Li M, Hakonarson H. ANNOVAR: functional annotation of genetic variants from high-throughput sequencing data. Nucleic Acids Research 2010 Sep;38:e164.
19. Zhang Z, Jung PP, Grouès V, May P, Linster C, Glaab E. Supporting data for "BSA4Yeast: Web-based QTL linkage analysis and bulk segregant analysis of yeast sequencing data"; GigaScience Database. 2019. http://dx.doi.orgxxxxxxxxxxxx.

Click here to access/download
Supplementary Material
bsa4yeast_supplementary_information.pdf
