# Supplementary material for: BSA4Yeast: Web-based quantitative trait locus linkage analysis and bulk segregant analysis of yeast sequencing data
Source: Gigascience. 2019 May 29;8(6):giz060. doi: 10.1093/gigascience/giz060 (PMC6571488; doi:10.1093/gigascience/giz060)
Supplement: giz060_Supplemental_File [file giz060_supplemental_file.pdf]

Supplementary Information

1.) File format descriptions (p. 1)

2.) BSA4Yeast - Experimental design, sequencing data processing and statistical methods (p. 2)

1.) File format descriptions

FASTQ format: The FASTQ file format is used to store nucleotide sequences and associated quality scores in a readable text format. In short, a typical FASTQ file uses the following four lines to encode a sequence: 1) Line 1 starts with the "@" character followed by a sequence identifier and an optional description. 2) Line 2 contains the original sequence. 3) Line 3 starts with a "+" character and optionally contains the same sequence identifier and description as in line 1. 4) Line 4 uses symbols to encode the quality values of the sequence in line 2 via ASCII-codes representing integer values. FASTQ files are often compressed and saved in the GNU zip format (an open source file compression program), with an additional .gz extension of the file name.

BAM format: The binary alignment map (BAM) file format contains the complete raw data from a DNA or RNA sequencing run. It uses a binary, lossless compression of the related uncompressed, text-based sequence alignment map (SAM) format in order to provide the raw data in a compact representation. More specifically, the BAM file consists of the following header and alignment sections: 1) The header describes general features of the complete file, e.g. the sample name, sample length, and alignment method. 2) The alignment section includes the read name, read sequence and quality, information about the alignment, and potential custom tags. Within the read name information about the chromosome, start coordinate, alignment quality, and match descriptor is encoded as a text string.

MAP format: The Map file format is the standard input format for the calculation of the G' statistic used to evaluate Bulk Segregant Analysis results. It uses a text-based format with the following four columns: The 1st column contains the name of the chromosome, the 2nd column specifies

the coordinates of the described genetic marker, the 3rd column lists the number of alleles which come from the 1st parent line, the 4th column contains the number of alleles from the 2nd parent line.

LENGTH format: The length file format is a file containing a list of chromosome lengths (in bp, one chromosome per line) and is used together with a corresponding map file (see above), when the user aims to plot the results of a Bulk Segregant QTL Analysis. The order of the chromosomes in the length file should be the same as the order in the map file.

2.) BSA4Yeast – Experimental design, sequencing data processing and statistical methods

Experimental design and sequencing data pre-processing
The genomic DNA from two parent lines (YO486 and YO502) and bulk segregant pools (high and low bulk in 2% glucose condition) were sequenced using 50-bp reads on an Illumina sequencer 2000. The approximate total numbers of reads for each condition were 5.7 M for YO486, 3.5 M for YO502, 10.6 M for high bulk of 2% glucose, 11.9 M for low bulk of 2% glucose, respectively. No bulk biological replicates were used. A next generation sequencing analysis pipeline was developed, which included quality control, preprocessing for short reads, mapping to the standard *S. cerevisiae* reference genome (UCSC release sacCer3; BWA, version: 0.7.4) (Li and Durbin 2009) and variant calling (SAMtools, version 0.1.18) (Li et al. 2009) using custom Python scripts. The average depth of coverage for bulk segregant pools was between 22x and 45x (see Table1 in the main manuscript). The genetic markers were defined as the nucleotides (SNPs) differing between haploid YO486 and YO502 strains, which had homogeneous calls with at depth of coverage of least 10x in the sequencing data. In total ~47k high-quality genetic markers (SNPs) were identified to differ between haploid YO486 and YO502 strains. To determine allele frequencies of all genetic markers (SNPs) in the pools at each genetic marker site identified, we estimated the counts of each allele in each segregant bulk pool using pileup files generated via SAMtools and custom scripts.

Statistical methods
The used approach for the analysis of allele frequencies in BSA-sequencing data sets was first proposed by Magwene *et al.* (2011). It considers two sources of variation in BSA-seq analyses. The first type of variation reflects the sampling variation for the segregants when creating the pools. The second type reflects variation resulting from the measurement approach, which includes variation in the library preparation, sequencing coverage, and alignment of reads, among

others. First, the allele frequencies are calculated for each sequencing bulk from the allele counts at the genetic markers sites. Using these allele frequencies, the method by Magwene *et al*. (2011) compares two sequencing bulks by accounting for the two sources of variation using the smoothed G statistic (G'). The G statistic has been reported to provide superior QTL mapping results than classical QTL estimation methods based on allelic frequency differences (Magwene *et al*., 2011). In particular, the G statistic can help to narrow QTL candidate intervals, since a decrease in the value of G is expected to indicate the causal site with high sensitivity. Moreover, the G statistic takes into account the strength of available experimental evidence, as determined by the sample size. By developing the G'-statistic as a smoothed version of the G-statistic, Magwene et al. use a weighted average of G across neighboring SNPs to remove noise associated with sequencing variation, making use of the fact that the real signal of divergence in allele frequency between bulks is conserved between closely linked sites, whereas random noise resulting from variable sequencing read coverage is not. The method can also take into account biological sample replicates to increase the accuracy of estimating the allele frequency of each bulk, thereby increasing the power of QTL analysis. Detailed definitions of the G and G' statistics and analyses of their properties are provided in the original publication by Magwene *et al*. (2011).

For the specific BSA-QTL analyses discussed here, for each pairwise comparison of sequencing bulks the G' statistic was used with a smoothing default window size of W = 33,750 kb. A false discovery rate (FDR) threshold of 0.05 was used to identify significant QTL regions. Next, a peak-calling algorithm was applied to identify peaks within these regions (Granek *et al.* 2013). We defined QTL regions as a continuous run of at least 10 genetic markers (SNPs) which cover at least 10 kb. Moreover, peaks were determined as contiguous sub-regions such that $G_i' = 0.90G_{max}'$, where G_{max}' is the site that has the largest G' within the QTL region (Granek *et al.* 2013). Finally, for each identified QTL peak with FDR < 0.05, candidate genes were identified based on the estimated G' statistic. The G' threshold corresponding to the chosen FDR cut-off of 0.05 is estimated automatically from the data.

References

Li, H., B. Handsaker, A. Wysoker, T. Fennell, J. Ruan et al., 2009 The Sequence Alignment/Map format and SAMtools. Bioinformatics 25: 2078–2079.

Magwene, P. M., J. H. Willis, and J. K. Kelly, 2011 The statistics of bulk segregant analysis using next generation sequencing. PLOS Comput. Biol. 7: e1002255.

Li, H., and R. Durbin, 2009 Fast and accurate short read alignment with Burrows-Wheeler transform. Bioinformatics 25: 1754–1760.

Granek, J. A., D. Murray, O. Kayrkci, and P. M. Magwene, 2013 The genetic architecture of biofilm formation in a clinical isolate of Saccharomyces cerevisiae. Genetics 193: 587–600.
