# Supplementary material for: BSA4Yeast: Web-based quantitative trait locus linkage analysis and bulk segregant analysis of yeast sequencing data
Source: Gigascience. 2019 May 29;8(6):giz060. doi: 10.1093/gigascience/giz060 (PMC6571488; doi:10.1093/gigascience/giz060)
Supplement: giz060_Response_to_Reviewer_Comments_Original_Submission [file giz060_response_to_reviewer_comments_original_submission.pdf]

# Author's Response To Reviewer Comments

Close

------------------------------
Reviewer #1 - Comments:
------------------------------

Zhang et al. developed a web-based interface allowing users to:
- Identify QTLs using sequencing data from a population of offsprings selected for extreme phenotype (called Bulk Segregant Analysis, BSA).
- Identify and annotate variants between the two S. cerevisiae parental strains, if the BSA has been done in S. cerevisiae species, to help the functional analysis of the QTL obtained.

The rational to provide this interface called BSA4Yeast is to allow lab with limited bioinformatic knowledge and resources to perform their own QTL analysis.

The paper is, for the most part, well written, and provides technical information in a clear and cohesive way. Furthermore, BSA4Yeast web-interface is also clear and user-friendly, which allows the users to perform the analysis and interpret the results.

However, I think some additional information is required to improve the usability and understanding of this method.

1.) In order for users with limited bioinformatic knowledge to understand the method, further information can be beneficial. For example, it should be explain what are .fastq, .bam, and .map files. Additionally, why is .vcf format not supported as well?

Response: We thank the reviewer for this useful suggestion and have added explanations of the .fastq, .bam, .map and .length-file formats to the Suppl. Mat. (page 1-2, referenced in the revised manuscript on page 2) and the BSA4Yeast web-page (https://bsa4yeast.lcsb.uni.lu, section "Usage & Formats"). Moreover, to explain why the vcf-format is not used, we have added the following text in the manuscript section "Materials and Methods/Functionality and Workflow" on page 2: "For the quality control analyses, the raw sequencing data is required, therefore we do not use files in the Variant Call Format (VCF) or the General Feature Format (GFF) as input. However, since VCF- and GFF-files represent an output derived from the analysis of raw sequencing data files, the user should in most cases also have access to corresponding raw fastq- or bam-files, which are both accepted as input."

2.) The authors do not describe the statistical analysis of the QTL in detail and further information is required to describe the G' statistic analysis, for example, explaining how the significant threshold is calculated and to which p-value/FDR it corresponds, and how replicate are used.

Response: To explain the statistical analyses and used parameters in more detail, we have added further information in the Supplementary Materials, referenced in the main manuscript on page 6 in section "Availability of supporting data and materials". The corresponding section covers the used QTL and G' statistics, the calculation of peaks depending on G', the chosen parameters including the used FDR threshold of 0.05, and how the method can use replicates to increase the statistical power. In the section "Experimental design and sequencing data pre-processing" in the Suppl. Mat. we also clarify that no bulk biological replicates were used in the presented example yeast BSA-QTL study.

3.) The authors also claim that BSA4Yeast can be used for BSA analysis in any species. However, this can be done only by providing .map files, which implies that the read mapping and allele count should be done by the user.
Hence the authors should:
-clarify early in the paper and in the abstract that BSA4Yeast is almost exclusively of BSA analysis in S. cerevisiae.
OR

-alter BSA4Yeast interface to support analysis of other species by allowing the users to provide their own reference genome. This is suggested in the discussion as a possible improvement, but it is unclear if the authors actually mean to implement it.

Response: To clarify this, in the abstract we have added the sentence "BSA4Yeast is mainly dedicated to bulk segregant analyses for S. cerevisiae, but we discuss possibilities for the experienced user to investigate data for other species based on the same software." In the manuscript on page 2, right column, we have also added the following more detailed explanation: "While the default setting of BSA4Yeast is to perform BSA-QTL analyses in yeast, the experienced user can also apply the software for QTL analyses on other species, either by adding further reference genomes to the source code implementation of BSA4Yeast (available at https://git-r3lab.uni.lu/zhi.zhang/bsa4yeast) or by creating a species-specific map file and chromosome length file as input, which requires a prior read mapping and allele count computation by the user. For adequate .map and .length input files, the public BSA4Yeast web-application can be used without further changes to identify QTLs for a chosen species of interest".

4.) It might be interesting if the authors test BSA4Yeast with .map files generated using other species to confirm that the analysis can deal with other genomes (for example: variability in the number of chromosomes from other species).

Response: We have checked whether further relevant datasets for BSA-QTL analyses were available on public data repositories, but could not identify a suitable dataset for another species. Therefore, we have created a new pseudo-map file with a different chromosome number than for the yeast genome, to verify the applicability of the software for other genome types. We successfully analyzed this pseudo-map file with the corresponding chromosome length file on BSA4Yeast. The corresponding pseudo-map file and pseudo-chromosome length file have been made available on the web-page (https://bsa4yeast.lcsb.uni.lu, section 'Examples' in the navigation bar on the left) and is mentioned in the revised manuscript on page 3, right column.

5.) BSA4Yeast requires variant information between the parental strains. The only way to do so is to provide the sequencing reads of the two parents. This needs to be indicated in the text.
Allowing the user to provide only a library of genetic positions if the cross have already been used and described (e.g. the RM*BY cross) might be a good feature to add.

Response: We have clarified this in the manuscript on page 2, right column, writing that "BSA4Yeast can be used for BSA-QTL analyses either if the sequencing reads of the parental lines are provided as input, or if the user only provides map-files without the original sequences (which however requires a prior manual analysis of the sequencing data by the user).". We think that the map-file format input is an alternative option to the input of the raw sequencing data that is similar to the suggested library of genetic positions, and has the benefit of using a standard file format that covers all necessary information for the implemented analyses.

6.) The authors indicated that BSA4Yeast will be updated and might be improved with new functionality. It would be good to indicate who will be in charge of maintaining the interface (ZZ or EG?). Will someone also be in charge of technical support for users? the methods there is no mention to the follow-up visit.

Response: A corresponding sentence has been added in the main manuscript on page 5, stating that: "The first author, Zhi Zhang, will continue to maintain the website interface, and provide technical support and updates for the users."

7.) Currently BSA4Yeast does not support compressed input (.fastq.gz), which might also be a good feature to add.

Response: We thank the reviewer for this useful suggestion, which has been implemented, and is mentioned in Section "Materials and Methods/Functionality and Workflow" on page 2, left column: "BSA4Yeast also supports compressed input in the fastq file format (.fastq.gz)."

------------------------------
Reviewer #2 - Comments:

------------------------------

The manuscript by Zhang et al, aims to describe a novel platform to perform linkage analysis and BSA using sequencing data. For this, the authors implement a series of tools into a single protocol to facilitate the identification of QTLs. This tool seems to be very useful and straightforward to utilize incorporating most of the variables considered when analyzing these large datasets. Even though this represents a great step forward towards simplifying this type of analysis, I have some minor comments that I believe should be addressed before the yeast community can use this interface:

1.) The example #1 provided was able to map two QTLs for cellular aging. Yet, no comparison is made with those results obtained by Jung et al, and whether results replicated, improved or were less informative. Further information on the dataset utilized (because there were several experiments performed on this paper) should be provided.

Response: We thank the reviewer for pointing this out and in our revised manuscript, we have added the following sentence in Section "Example application" on page 3 to clarify this: "To test BSA4Yeast, we re-analysed one dataset from Jung et al. (2018), which consists of the genomic DNA from two parent lines (YO486 and YO502) and two bulk segregant pools (each bulk comprising the 50 segregants with the highest or shortest chronological life span under low glucose conditions) sequenced using 50-bp reads on an Illumina sequencer 2000 (see the Suppl. Mat. for further details). With this data, the BSA4Yeast software recomputes and replicates the QTL results previously published by Jung et al. (2018)."

2.) Detailed information on the quality options at each step should be provided.

Response: We have added the following text on page 2, right column, of the manuscript to address this point: "Although BSA4Yeast cannot provide detailed quality information for each analysis step due to the wide range of possible noise sources (experimental and technical), the multitude of quality metrics for sequencing data and their data-dependent interpretation, if a job is not successfully complete, the software will show a guide label (yellow) on the job status page, which provides the user with indications on how to adjust the parameters. However, we also recommend that users perform their own quality control analyses for their data before uploading it on the web-interface."

3.) Based on the description, is not clear to me how non-cerevisiae organisms can be studied using this interface. Have the authors considered the possibility of uploading GFF files to facilitate the study of other species?

Response: BSA4Yeast does not support the GFF format as input, and in order to explain this, we have added the following comment in Section "Materials and Methods/Functionality and Workflow" on page 2, right column (see also the comment to question 1 by reviewer 1): "For the quality control analyses the raw sequencing data is required, therefore we do not use files in the Variant Call Format (VCF) or the General Feature Format (GFF) as input. However, since VCF- and GFF-files represent an output derived from the analysis of raw sequencing data files, the user should typically also have access to corresponding raw fastq- or bam-files, which are both accepted as input." Moreover, to clarify how other species can be analysed, on page 2, right column, we have added the following more detailed explanation (see also the comment to question 3 by reviewer 1): "While the default setting of BSA4Yeast is to perform BSA-QTL analyses in yeast, the experienced user can also apply the software for QTL analyses on other species, either by adding further reference genomes to the source code implementation of BSA4Yeast (available at https://git-r3lab.uni.lu/zhi.zhang/bsa4yeast) or by creating a species-specific map file and chromosome length file as input, which requires a prior read mapping and allele count computation by the user. For adequate .map and .length input files, the public BSA4Yeast web-application can be used without further changes to identify QTLs for a chosen species of interest".

Close
