# Supplementary material for: BSA4Yeast: Web-based quantitative trait locus linkage analysis and bulk segregant analysis of yeast sequencing data
Source: Gigascience. 2019 May 29;8(6):giz060. doi: 10.1093/gigascience/giz060 (PMC6571488; doi:10.1093/gigascience/giz060)
Supplement: giz060_Response_to_Reviewer_Comments_Revision_1 [file giz060_response_to_reviewer_comments_revision_1.pdf]

Author's Response To Reviewer Comments

Close

Dear Editor,

we thank you and the reviewers for the efficient handling of the manuscript. We have addressed the three final editorial issues mentioned in the response letter as follows:

1) GigaDB repository: We have included a citation of the upcoming GigaDB dataset (reference 19) - please note that the DOI number was not yet available, but we have been informed by Chris Armit that the GigaScience Editors will add the final DOI number to template DOI link in our manuscript.

2) All highlighting / tracking of changes for the reviewers has been removed from the final manuscript.

3) Our software application has been registered in the SciCrunch.org database and the RRID has been added to the manuscript. (the RRID-link is expected to become active after the 20th of April).

Yours sincerely,
Enrico Glaab

Close
