# Supplementary material for: BSA4Yeast: Web-based quantitative trait locus linkage analysis and bulk segregant analysis of yeast sequencing data
Source: Gigascience. 2019 May 29;8(6):giz060. doi: 10.1093/gigascience/giz060 (PMC6571488; doi:10.1093/gigascience/giz060)
Supplement: giz060_Response_to_Reviewer_Comments_Revision_2 [file giz060_response_to_reviewer_comments_revision_2.pdf]

Author's Response To Reviewer Comments

Close

Dear Editor,

many thanks for the quick processing of the manuscript and the finalization of the GigaDB entry. In the revised version, I have inserted the final doi link (http://gigadb.org/dataset/100595) in reference 19, as suggested.

Best regards,
Enrico Glaab

Close
