# Supplementary material for: BSA4Yeast: Web-based quantitative trait locus linkage analysis and bulk segregant analysis of yeast sequencing data
Source: Gigascience. 2019 May 29;8(6):giz060. doi: 10.1093/gigascience/giz060 (PMC6571488; doi:10.1093/gigascience/giz060)
Supplement: giz060_Reviewer_1_Report_Original_Submission -- Christian Brion, Ph.D, — 12/8/2018 Reviewed [file giz060_reviewer_1_report_original_submission.pdf]

Reviewer Report

Title: BSA4Yeast: Web-based QTL linkage analysis and bulk segregant analysis of yeast sequencing data

Version: Original Submission **Date:** 12/8/2018

Reviewer name: Christian Brion, Ph.D,

Reviewer Comments to Author:

Zhang et al. developed a web-based interface allowing users to:
- Identify QTLs using sequencing data from a population of offsprings selected for extreme phenotype (called Bulk Segregant Analysis, BSA).
- Identify and annotate variants between the two S. cerevisiae parental strains, if the BSA has been done in S. cerevisiae species, to help the functional analysis of the QTL obtained.

The rational to provide this interface called BSA4Yeast is to allow lab with limited bioinformatic knowledge and resources to perform their own QTL analysis.

The paper is, for the most part, well written, and provides technical information in a clear and cohesive way. Furthermore, BSA4Yeast web-interface is also clear and user-friendly, which allows the users to perform the analysis and interpret the results.

However, I think some additional information is required to improve the usability and understanding of this method.

1) In order for users with limited bioinformatic knowledge to understand the method, further information can be beneficial. For example, it should be explain what are .fastq, .bam, and .map files. Additionally, why is .vcf format not supported as well?

2) The authors do not describe the statistical analysis of the QTL in detail and further information is required to describe the G' statistic analysis, for example, explaining how the significant threshold is calculated and to which p-value/FDR it corresponds, and how replicate are used.

3) The authors also claim that BSA4Yeast can be used for BSA analysis in any species. However, this can be done only by providing .map files, which implies that the read mapping and allele count should be done by the user.

Hence the authors should:

-clarify early in the paper and in the abstract that BSA4Yeast is almost exclusively of BSA analysis in S. cerevisiae.

OR

-alter BSA4Yeast interface to support analysis of other species by allowing the users to provide their own reference genome. This is suggested in the discussion as a possible improvement, but it is unclear if the authors actually mean to implement it.

4) It might be interesting if the authors test BSA4Yeast with .map files generated using other species to confirm that the analysis can deal with other genomes (for example: variability in the number of chromosomes from other species).

5) BSA4Yeast requires variant information between the parental strains. The only way to do so is to

provide the sequencing reads of the two parents. This needs to be indicated in the text.
Allowing the user to provide only a library of genetic positions if the cross have already been used and described (e.g. the RM*BY cross) might be a good feature to add.
6) The authors indicated that BSA4Yeast will be updated and might be improved with new functionality. It would be good to indicate who will be in change of maintaining the interface (ZZ or EG?). Will someone also be in charge of technical support for users?
7) Currently BSA4Yeast does not support compressed input (.fastq.gz), which might also be a good feature to add.

Level of Interest

Please indicate how interesting you found the manuscript: Choose an item.

Quality of Written English

Please indicate the quality of language in the manuscript: Choose an item.

Declaration of Competing Interests

Please complete a declaration of competing interests, considering the following questions:

- Have you in the past five years received reimbursements, fees, funding, or salary from an organisation that may in any way gain or lose financially from the publication of this manuscript, either now or in the future?
- Do you hold any stocks or shares in an organisation that may in any way gain or lose financially from the publication of this manuscript, either now or in the future?
- Do you hold or are you currently applying for any patents relating to the content of the manuscript?
- Have you received reimbursements, fees, funding, or salary from an organization that holds or has applied for patents relating to the content of the manuscript?
- Do you have any other financial competing interests?
- Do you have any non-financial competing interests in relation to this paper?

If you can answer no to all of the above, write 'I declare that I have no competing interests' below. If your reply is yes to any, please give details below.

I declare that I have no competing interests

I agree to the open peer review policy of the journal. I understand that my name will be included on my report to the authors and, if the manuscript is accepted for publication, my named report including any attachments I upload will be posted on the website along with the authors' responses. I agree for my report to be made available under an Open Access Creative Commons CC-BY license

(http://creativecommons.org/licenses/by/4.0/). I understand that any comments which I do not wish to be included in my named report can be included as confidential comments to the editors, which will not be published.

I agree to the open peer review policy of the journal

To further support our reviewers, we have joined with Publons, where you can gain additional credit to further highlight your hard work (see: https://publons.com/journal/530/gigascience). On publication of this paper, your review will be automatically added to Publons, you can then choose whether or not to claim your Publons credit. I understand this statement.

Yes Choose an item.
