# Supplementary material for: BSA4Yeast: Web-based quantitative trait locus linkage analysis and bulk segregant analysis of yeast sequencing data
Source: Gigascience. 2019 May 29;8(6):giz060. doi: 10.1093/gigascience/giz060 (PMC6571488; doi:10.1093/gigascience/giz060)
Supplement: giz060_Reviewer_2_Report_Original_Submission -- Francisco Cubillos — 12/11/2018 Reviewed [file giz060_reviewer_2_report_original_submission.pdf]

Reviewer Report

Title: BSA4Yeast: Web-based QTL linkage analysis and bulk segregant analysis of yeast sequencing data

Version: Original Submission **Date:** 12/11/2018

Reviewer name: Francisco Cubillos

Reviewer Comments to Author:

The manuscript by Zhang et al, aims to describe a novel platform to perform linkage analysis and BSA using sequencing data. For this, the authors implement a series of tools into a single protocol to facilitate the identification of QTLs. This tool seems to be very useful and straightforward to utilize incorporating most of the variables considered when analyzing these large datasets. Even though this represents a great step forward towards simplifying this type of analysis, I have some minor comments that I believe should be addressed before the yeast community can use this interface:
1. The example #1 provided was able to map two QTLs for cellular aging. Yet, no comparison is made with those results obtained by Jung et al, and whether results replicated, improved or were less informative. Further information on the dataset utilized (because there were several experiments performed on this paper) should be provided.
2. Detailed information on the quality options at each step should be provided.
3. Based on the description, is not clear to me how non-cerevisiae organisms can be studied using this interface. Have the authors considered the possibility of uploading GFF files to facilitate the study of other species?

Level of Interest

Please indicate how interesting you found the manuscript: Choose an item.

Quality of Written English

Please indicate the quality of language in the manuscript: Choose an item.

Declaration of Competing Interests

Please complete a declaration of competing interests, considering the following questions:

- Have you in the past five years received reimbursements, fees, funding, or salary from an organisation that may in any way gain or lose financially from the publication of this manuscript, either now or in the future?
- Do you hold any stocks or shares in an organisation that may in any way gain or lose financially from the publication of this manuscript, either now or in the future?

- Do you hold or are you currently applying for any patents relating to the content of the manuscript?
- Have you received reimbursements, fees, funding, or salary from an organization that holds or has applied for patents relating to the content of the manuscript?
- Do you have any other financial competing interests?
- Do you have any non-financial competing interests in relation to this paper?

If you can answer no to all of the above, write 'I declare that I have no competing interests' below. If your reply is yes to any, please give details below.

I declare that I have no competing interests

I agree to the open peer review policy of the journal. I understand that my name will be included on my report to the authors and, if the manuscript is accepted for publication, my named report including any attachments I upload will be posted on the website along with the authors' responses. I agree for my report to be made available under an Open Access Creative Commons CC-BY license (http://creativecommons.org/licenses/by/4.0/). I understand that any comments which I do not wish to be included in my named report can be included as confidential comments to the editors, which will not be published.

I agree to the open peer review policy of the journal

To further support our reviewers, we have joined with Publons, where you can gain additional credit to further highlight your hard work (see: https://publons.com/journal/530/gigascience). On publication of this paper, your review will be automatically added to Publons, you can then choose whether or not to claim your Publons credit. I understand this statement.

Yes Choose an item.
